# The Treatment of Gall Stone, with Cases*Abstract of a paper read at the fourth annual meeting of the Southern Surgical and Gynecological Association.

**Published:** 1892-04

**Authors:** W. E. B. Davis

**Affiliations:** Rome, Ga.; President of the Tri-State Medical Society of Alabama, Georgia, and Tennessee; Secretary of the Southern Surgical and Gynecological Association, Fellow of the American Association of Obstetricians and Gynecologists; Honorary member of the Medical Society of the State of New York; Formerly member of the Jefferson County (Birmingham) Board of Medical Examiners, etc., etc., etc.


### THE TREATMENT OF GALL STONES, WITH CASES.*

BY W. E. B. DAVIS, M. D., ROME, GA.

President of the Tri-State Medical Society of Alabama, Georgia, and Tennessee; Secretary of the Southern Surgical and Gynecological Association, Fellow of the American Association of Obstetricians and Gynecologists; Honorary member of the Medical Society of the State of New York; Formerly member of the Jefferson County (Birmingham) Board of Medical Examiners, etc., etc., etc.

He said:—The treatment during the attacks, consisted in hypodermics of morphine and atropine, with the use of ether and chloroform until the other remedies have had time to take effect. It is usually soothing to place the patient in a hot bath; and large draughts of hot water will relieve the distressing nausea. After gall-stones have formed, experience does not warrant us in placing confidence in medical treatment for their cure. The sweet oil draughts, as has been abundantly shown, only become saponified and give rise to stone-like masses. Turpentine, chloroform and wild yam are not curative. Perhaps something may yet be found that, when injected into the bladder, will dissolve the stones. This is especially desirable for stones located in the ducts.

It would seem that medical treatment would prevent their formation, but so far, there is no very good evidence to show that any medicine has this effect.

A stone in the gall-bladder produces such a condition as to favor the formation of other stones; and after the operation for the removal of the stone, and the relief of the local condition, we have no return of the disease, except in a small per cent. of cases. This is so in those cases in which stones had been forming rapidly before the operation, and would go far to show the importance of the local causes of the disease. Then in addition to general tonics, iron, phosphate of soda, mineral waters, etc., our dependence must be placed on operative procedures.

In some cases it may be difficult, or even impossible, to

(Abstract of a paper read at the fourth annual meeting of the Southern Surgical and Gynecological Association.)

make a diagnosis of gall-stones, but it has been said very correctly that the mistake is much oftener made in calling gall-stones something else than in calling something else gall-stones. Paroxysms of epigastric pain, with tenderness over the lower hepatic region, accompanied with bile in the urine, and followed by clay colored stools, and sometimes the passage of stones, are symptoms on which dependence must be placed. The shoulder pain is rarely present and jaundice is most frequently absent. It is only when there is obstruction in the hepatic or common duct that this symptom is to be expected; and often the obstruction is so evanescent as not to give rise to sufficient obstruction to produce jaundice.

Where there are frequent attacks of biliary colic, it is best to operate and give the patient the benefit of the exploration, and avoid the dangers of peritonitis. It is not conservatism to delay operation where there are obstructive symptoms, until the liver has become involved and the patient's blood poisoned. He had seen a number of these neglected cases in which an operation could afford no chance whatever. He reported a case of death from peritonitis following repeated attacks of biliary colic, where there was sufficient warning to save the patient, but her physician would not advise an operation. In some cases, however, there are no symptoms to indicate the presence of a stone, until peritonitis has resulted from ulceration thus induced. During the past month he had operated on such a case at Asheville, Ala., for Dr. D. E. Cason. The patient was 74 years of age and had never experienced any symptoms of gall-stones. He recommends cholecystotomy, and opens the bladder and sutures it to the parietes at one operation. He reported a case in which he removed 51 gall-stones from the bladder, one of them being impacted in the cystic duct, and the patient made a perfect recovery.

Cholecystectomy, the removal of the gall-bladder, should never be an operation of selection and only resorted to when cholecystotomy is not possible. Do not stitch the bladder to the parietes and wait for adhesion before opening the viscus, as it is necessary for it to be opened and emptied before the abdominal wound is closed, in order to recognize conditions which will require manipulations within the abdomen as well as within the bladder. Stones impacted in the duct must be dislodged and pushed into the bladder or duodenum. It may be necessary to break them up with a needle before this is possible. In some cases the duct should be incised and sutured. When the obstruction in the common duct cannot be relieved, cholecysto-enterostomy should be resorted to.

